# Kahweol, a Diterpenoid Molecule, Inhibits CTGF-Dependent Synthetic Phenotype Switching and Migration in Vascular Smooth Muscle Cells

**DOI:** 10.3390/molecules26030640

**Published:** 2021-01-26

**Authors:** Jeong Hee Lee, Seok Tae Choi, Young Jin Kang

**Affiliations:** 1 Department of Pharmacology, College of Medicine, Yeungnam University, 170 Hyunchung-Ro, Nam-Gu, Daegu 42415, Korea; jeonghee03@naver.com; 2 Department of Microbiology, Yeungnam University, 170 Hyunchung-Ro, Nam-Gu, Daegu 42415, Korea; tjrxopk@naver.com

**Keywords:** kahweol, connective tissue growth factor, vascular smooth muscle cell, synthetic phenotype switching

## Abstract

Vascular smooth muscle cell (VSMC) phenotype switching from contractile to synthetic is essential for proliferation and migration in vascular pathophysiology. Connective tissue growth factor (CTGF) is a matricellular protein involved in cell adhesion, migration, and proliferation. Kahweol, a diterpene molecule in arabica coffee beans, has been reported to have anti-inflammatory, antiproliferative, and apoptotic effects in many cells. However, in VSMCs, the effects of kahweol on CTGF activities have not been investigated. Thus, in this study, the effects and associated mechanisms of kahweol in CTGF-dependent phenotype switching and migration in VSMCs were examined. Experiments were performed on primary rat aortic smooth muscle cells and a rat VSMC line, A7r5. Western blot analysis was used to determine the protein levels. The mRNA levels of synthetic markers were measured by qRT-PCR. Migration of VSMCs was evaluated by wound healing and transwell assays. Kahweol reduced the angiotensin II (Ang II)-induced CTGF expression. Further, kahweol inhibited expressions of synthetic phenotype markers of VSMC. The kahweol-reduced synthetic marker protein levels were reversed by the administration of rCTGF. However, expressions of contractile phenotype markers of VSMC were not affected. Kahweol suppressed Ang II-stimulated VSMC migration. Moreover, kahweol downregulated Ang II-induced p-FAK, p-Erk, and Yes-associated protein (YAP) protein expressions. Taken together, in Ang II-stimulated VSMCs, kahweol inhibited CTGF-dependent synthetic phenotype switching and migration, with focal adhesion kinase (FAK), Erk, and YAP involved in the underlying mechanisms of the kahweol effects. These results suggest that kahweol has a potential as a therapeutic agent to inhibit CTGF, which is a molecular target in sclerogenic vascular disease.

## 1. Introduction

The prevalence of metabolic syndrome is a global trend, and the incidence of cardiovascular diseases, including atherosclerosis, a complication of metabolic syndrome, is steadily increasing [1,2]. Atherosclerosis is a chronic inflammatory disease in which the internal diameter of arteries is reduced by atheromatous plaques, resulting in myocardial infarction and stroke [1]. Therefore, atherosclerosis is an independent and high risk factor for life-threatening cardiovascular disease. Treatments for atherosclerosis are based on controlling risk factors such as obesity, hypertension, hyperglycemia, and hyperlipidemia [3]. However, these treatments are limited in preventing the molecular causes involved in the development of atherosclerosis.

Vascular smooth muscle cells (VSMCs), which have a crucial role in the development of atherosclerosis, are mainly distributed in the media layer of blood vessels and have a characteristic that allows them to reversibly change into contractile or synthetic phenotypes depending on the cellular microenvironments [4,5,6]. In the development of atherosclerosis, endothelial, and immune cells release various stimulating factors, including angiotensin II (Ang II) that induces synthetic VSMCs. Synthetic VSMCs are able to migrate from the media layer to the intima, and migrated VSMCs produce extracellular matrix (ECM) proteins in the intima, which results in increased artery wall thickness and stiffness [4,5,6,7,8]. It has been reported that Ang II and platelet-derived growth factor (PDGF), which induce synthetic phenotype switching in VSMCs, increase VSMC migration via phosphorylation of focal adhesion kinase (FAK) [9,10,11]. Because regulation of synthetic VSMCs has pivotal roles in the pathophysiology of atherosclerosis, it may be a key target for cardiovascular interventions.

Coffee beans contain thousands of compounds, and one of which is kahweol, a coffee diterpene molecule that is abundant in oil derived from arabica coffee beans. Recent studies have reported that kahweol has various physiological activities such as antiproliferative [12], anti-tumor [12,13], anti-inflammatory [14,15], antiangiogenic [14], antifibrotic [16], and antioxidant [17] effects in many cell types. So far, most kahweol studies have reported on its anti-inflammatory effect in the liver [14,15] or its anti-tumor effect in cancer [12,13]. It has been reported that kahweol ameliorates liver inflammation by inhibiting nuclear factor kappa B (NF-κB) and signal transducer and transcription (STAT3) activation in hepatocytes [15]. It also has been reported that kahweol induces apoptosis by inhibiting phosphorylation of extracellular signal regulated kinase (Erk) in HER2-overexpressing breast cancer cells [12]. Nevertheless, the protective effects of kahweol on vascular diseases, including atherosclerosis, have not been sufficiently demonstrated.

Connective tissue growth factor (CTGF), also known as CCN2, is a matricellular protein of the CCN family (CCN1–6) and is involved in intercellular signaling regulations. Structurally, CTGF is consist of four modules, the IGFBP (insulin-like growth factor binding protein), VWC (Von Willebrand type C repeat), TSP1 (thrombospondin type 1 receptor), and cysteine-knot module. Previously, this 38 kDa protein is well recognized as a mediator of fibrosis [18,19,20,21,22]. CTGF is also involved in proliferation, migration, chemotaxis, and ECM production by which it contributes to the development of atherosclerosis [19,20,22]. For example, CTGF induces fibroblast adhesion via binding to fibronectin, which activates FAK phosphorylation and enhances focal adhesion formation and migration [23,24]. Several studies have demonstrated that Ang II [11], thrombin [25], and advanced glycation end products (AGE) [26] induce CTGF expression in VSMCs. Previously, CTGF was reported to be overexpressed in atherosclerotic plaques [27], and to stimulate VSMC proliferation and migration [28]. Furthermore, it was demonstrated that CTGF contributes to the phenotypic switching observed in oxidative stress-stimulated VSMCs [29]. Although kahweol is reported to decrease hepatic fibrosis by inhibiting the expression of CTGF [16], in VSMCs, the effects of kahweol on CTGF expression and VSMC phenotype switching remain undescribed. In the present study, the effects of kahweol on CTGF expression, VSMC phenotype switching, and the underlying mechanisms were investigated.

## 2. Results

### 2.1. Kahweol Reduces Ang II-Induced CTGF Protein Expression in VSMCs

**Figure 1:**
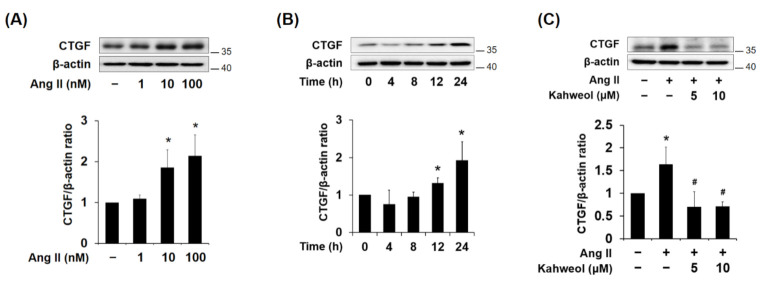
Effect of kahweol on connective tissue growth factor (CTGF) protein expression in Ang II-stimulated vascular smooth muscle cells (VSMCs). (**A**,**B**) Western blot analysis of CTGF in Ang II-treated VSMCs. Cells were exposed to various concentrations of Ang II (0, 1, 10, and 100 nM) for 24 h. In addition, cells were treated with Ang II 100 nM for the indicated times (0, 4, 8, 12, and 24 h). Protein samples were refined from cultured VSMCs treated with Ang II. (**C**) Cells were pretreated in the presence or absence of kahweol (0, 5, and 10 μM) for one hour, and then stimulated with 100 nM Ang II for a further 24 h. Protein levels of CTGF and β-actin were determined by Western blotting with specific antibodies. Bar graphs present the densitometric quantification of the Western blot bands. Results are representative of three independent experiments. *, *p* < 0.05 vs. untreated control; #, *p* < 0.05 vs. Ang II group.

To determine whether Ang II induces CTGF expression in VSMCs, the cells were treated with various concentrations of Ang II (1, 10, and 100 nM) for 24 h. Separately, cells were treated with 100 nM Ang II for various times (0, 4, 8, 12, and 24 h). Ang II treatment increased CTGF protein expression in dose- and time-dependent manners (Figure 1A,B). Next, to investigate whether kahweol suppresses CTGF expression in VSMCs, various doses of kahweol were treated to Ang II-stimulated VSMCs. Kahweol reduced CTGF expression by half relative to the Ang II treatment group (Figure 1C). To investigate whether kahweol affects cell viability, kahweol (5 and 10 μM) was treated in VSMCs. In MTT assay, cell viability was not significantly affected at 5 and 10 μM of kahweol (data not shown). These results indicate that kahweol suppresses Ang II-induced CTGF expression in VSMCs.

### 2.2. Kahweol Inhibits Ang II-Induced CTGF and Synthetic Phenotype Marker Expressions, and rCTGF Reverses the Kahweol Effect in VSMCs

**Figure 2:**
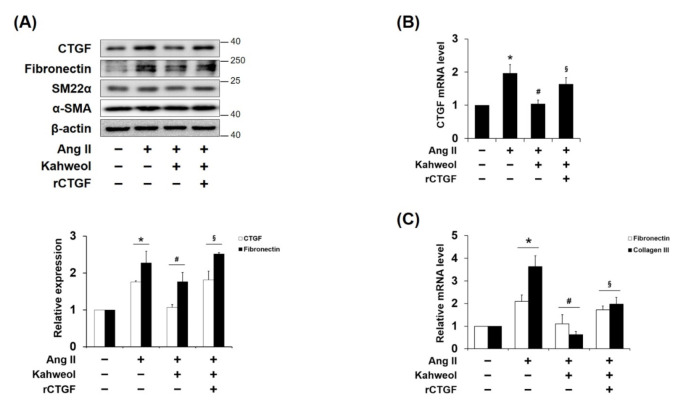
Kahweol effect on phenotype markers in Ang II-stimulated VSMCs. Cells were pretreated with rCTGF (50 ng/mL) and kahweol (10 μM) at one hour intervals and then stimulated with Ang II for 24 h. (**A**) Protein levels of CTGF, fibronectin, SM22α, α-SMA, and β-actin were determined by Western blotting with specific antibodies. Bar graphs present the densitometric quantification of the Western blot bands. (**B**,**C**) mRNA levels of CTGF, fibronectin, and collagen III were determined by qRT-PCR analysis. Results are representative of three independent experiments. *, *p* < 0.05 vs. untreated control; #, *p* < 0.05 vs. Ang II group; §, *p* < 0.05 vs. kahweol + Ang II treated group.

VSMC phenotype switching has pivotal roles in the pathophysiology of atherosclerosis [4,5,6,7,8]. Thus, whether kahweol regulates the protein expressions of synthetic phenotype marker, such as fibronectin, and contractile markers, such as SM22α and α-SMA, were investigated. Ang II-induced fibronectin was reduced by kahweol, and rCTGF administration restored it. Interestingly, the expression of the SM22α and α-SMA contractile markers were not significantly altered by kahweol treatment (Figure 2A). Additionally, the mRNA levels of CTGF and synthetic markers were investigated. Kahweol inhibited Ang II-induced mRNA levels of fibronectin and collagen III as it did for CTGF expression, and rCTGF administration reversed the kahweol effect (Figure 2B,C). These data suggest that kahweol inhibits synthetic phenotype switching.

### 2.3. Kahweol Inhibits Ang II-Induced FAK and Erk Phosphorylation and Migration, and rCTGF Reverses the Kahweol Effects in VSMCs

**Figure 3:**
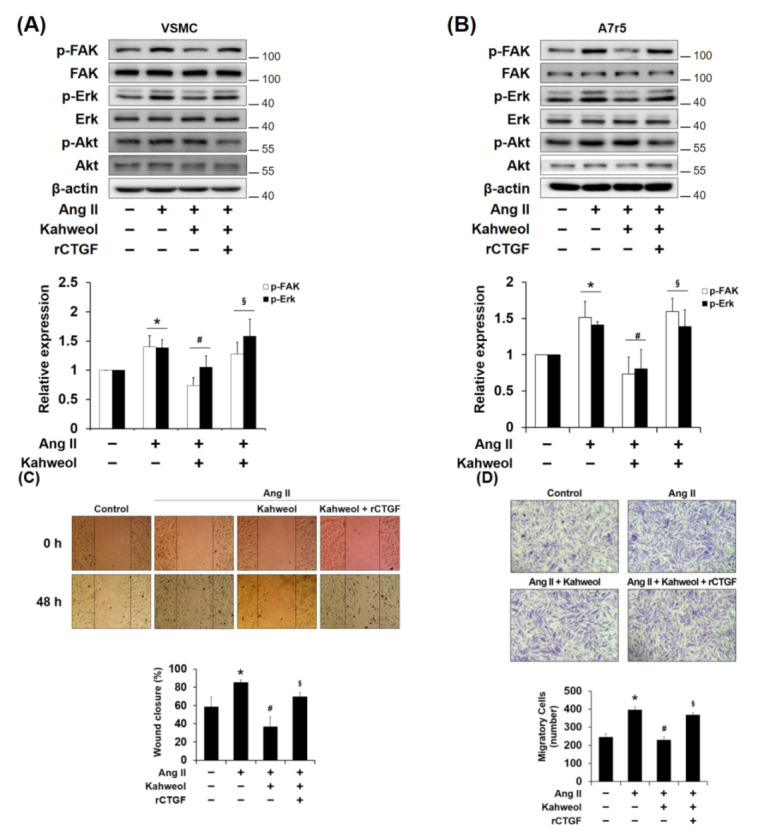
The involvement of focal adhesion kinase (FAK) and Erk in the mechanism underlying the kahweol effect on migration in Ang II-stimulated VSMCs. (**A**,**B**) Cells were pretreated with rCTGF and kahweol at one hour intervals and then stimulated with Ang II for 24 h. Protein levels of phosphorylated FAK, Erk, Akt, and β-actin were determined by Western blotting with specific antibodies. Bar graphs present the densitometric quantification of the Western blot bands. (**C**) Cells were scratched with a micropipette tip to form a cell- free (wounded) area and pretreated with rCTGF and kahweol at one hour intervals and then stimulated with Ang II for 48 h. Wound areas were visualized using a phase-contrast microscope. The distance migrated was determined as the average of the distances of each cell from the wound boundary. (**D**) For selective migration assay, a transwell assay was performed. Cells were seeded on the inner chamber under the same treatment condition, as in Figure 3C. After fixing, cells were visualized by crystal violet staining. Unmigrated cells were scraped off, and the migrated cells were counted under a light microscope. Results are representative of three independent experiments. *, *p* < 0.05 vs. untreated control; #, *p* < 0.05 vs. Ang II group; §, *p* < 0.05 vs. kahweol + Ang II treated group.

To investigate the underlying mechanisms that contribute to the regulation of CTGF by kahweol, the levels of phosphorylated Erk, FAK, and protein kinase B (Akt), which have been reported to mediate VSMC migration and proliferation [9], were observed in primary cultured VSMCs (rat origin cells) and A7r5 (rat origin cell line). Kahweol inhibited Ang II-induced FAK and Erk phosphorylation, but there was no significant effect on Akt phosphorylation. The decreases in FAK and Erk phosphorylations were restored by rCTGF administration (Figure 3A,B). Next, the kahweol effect on migration in Ang II-stimulated VSMCs was evaluated to assess wound healing effects. Ang II-stimulated VSMCs showed approximately wound healing over 85% of the scratched area compared to that of the untreated control, which showed wound healing over 58% of the scratched area. Kahweol treatment inhibited the wound healing to 36% of the scratched area while rCTGF administration restored it to 70% (Figure 3C). Furthermore, an inhibitory effect of kahweol on VSMC migration was also observed in the additionally performed transwell analysis. Ang II increased migration by 1.6-fold relative to the untreated control. Migration was reduced to the control level by kahweol treatment, and rCTGF administration significantly reversed migration, increasing it by 1.5-fold (Figure 3D). Taken together, these results indicate that kahweol inhibits Ang II-induced FAK and Erk phosphorylation and cell migration.

### 2.4. Kahweol Inhibits Fibronectin Expression by Reducing CTGF Expression in VSMCs

**Figure 4:**
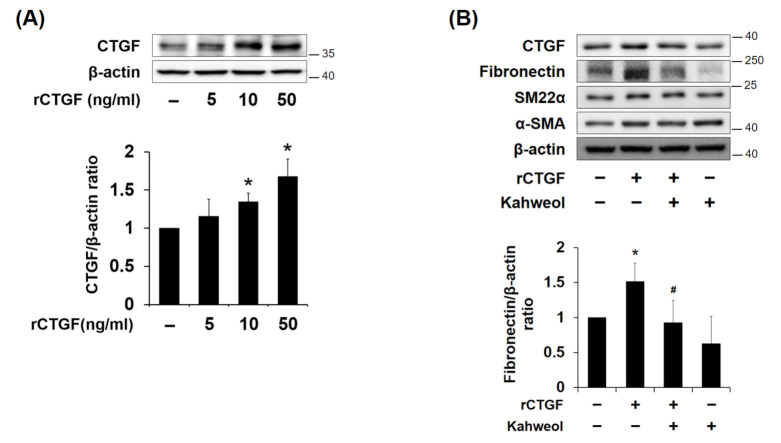
Effect of kahweol on CTGF induced phenotype switching in VSMCs. (**A**) Cells were stimulated with rCTGF (50 ng/mL) for 24 h at the indicated dosages and then (**B**) Cells were pretreated with rCTGF (50 ng/mL) for one hour and then treated with kahweol (10 μM) for 24 h. Protein levels of CTGF, fibronectin, SM22α, α-SMA, and β-actin were determined by Western blotting with specific antibodies. Bar graphs present the densitometric quantification of the Western blot bands. Results are representative of three independent experiments. *, *p* < 0.05; vs. untreated control; #, *p* < 0.05; vs. rCTGF group.

To investigate whether kahweol inhibits synthetic markers via CTGF regulation, CTGF expression was induced by rCTGF administration. The cells were treated with different concentrations of rCTGF (5, 10, and 50 ng/mL), and a significant increase in CTGF occurred at 50 ng/mL. rCTGF increased CTGF expression by 1.6-fold relative to the untreated control. (Figure 4A). Therefore, 50 ng/mL rCTGF was used in the following experiments. Fibronectin expression was increased by rCTGF administration, but it was suppressed by kahweol treatment. The SM22α and α-SMA expressions were not affected. (Figure 4B). The inhibitory effect of kahweol on CTGF and fibronectin expressions were consistent with the previously observed result shown in Figure 2. These results indicate that kahweol inhibits synthetic marker expression by regulating CTGF.

### 2.5. Kahweol Inhibits CTGF-Induced FAK Phosphorylation and Migration in VSMCs

**Figure 5:**
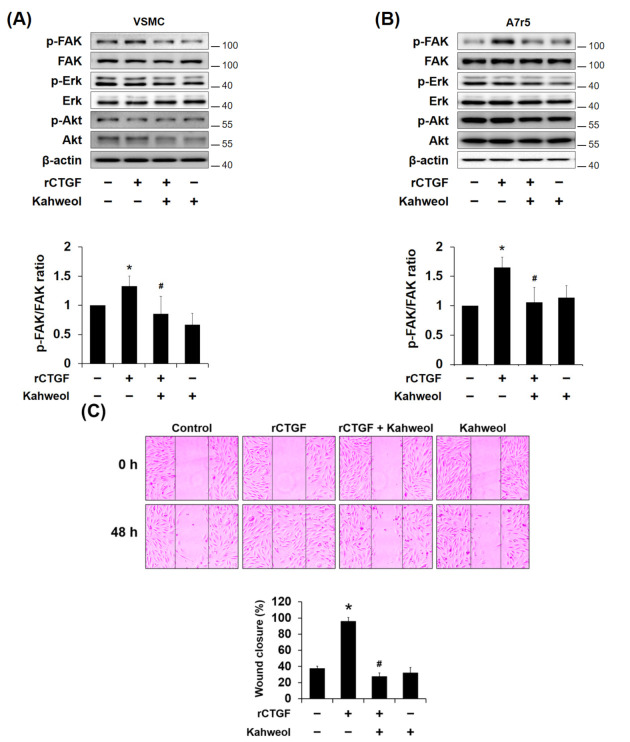
The involvement of FAK in the mechanism underlying the kahweol effect on migration in CTGF-stimulated VSMCs. (**A**,**B**) Cells were pretreated with rCTGF (50 ng/mL) for one hour and then treated with kahweol (10 μM) for 24 h. Soluble lysates were subjected to Western blotting for FAK, Erk, and Akt. Protein levels of phosphorylated FAK, Erk, Akt, and β-actin were determined by Western blotting with specific antibodies. Bar graphs present the densitometric quantification of the Western blot bands. (**C**) Cells were scratched with a micropipette tip to form a cell-free (wounded) area and pretreated with rCTGF (50 ng/mL) for one hour and then treated with kahweol (10 μM) for for 48 h. Wound areas were visualized using a phase-contrast microscope. The distance migrated was determined as the average of the distances of each cell from the wound boundary. Results are representative of three independent experiments. *, *p* < 0.05 vs. untreated control; #, *p* < 0.05 vs. rCTGF group.

To confirm the underlying mechanisms that contribute to the regulation of CTGF by kahweol, the levels of phosphorylated Erk, FAK, and protein kinase B (Akt) have been investigated. Kahweol inhibited rCTGF-induced FAK phosphorylation, whereas Erk and Akt phosphorylation were not affected (Figure 5A,B). Additionally, to determine whether VSMC migration is CTGF dependent, cells were stimulated with rCTGF. While the untreated control cells showed 37% of the scratched area underwent wound healing, rCTGF administration increased the area with wound healing to 95%, while kahweol inhibited wound healing to only 28% of the scratched area (Figure 5C). Taken together, these results indicate that kahweol inhibits CTGF-dependent FAK phosphorylation and VSMC migration.

### 2.6. Kahweol Inhibits Ang II-Induced YAP Expression, and rCTGF Reverses the Kahweol Effect in VSMCs

**Figure 6:**
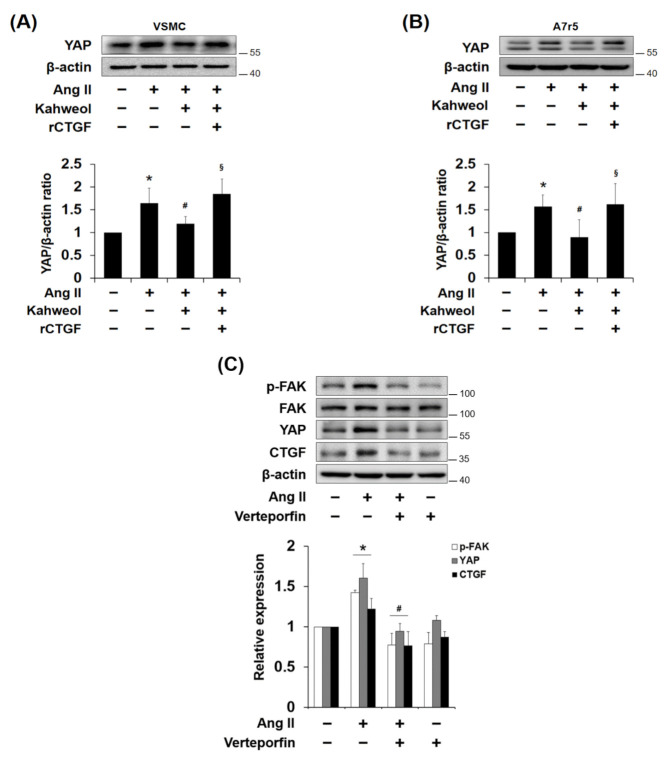
The involvement of Yes-associated protein (YAP) in the regulatory effect of kahweol in Ang II-stimulated VSMCs. (**A**,**B**) Cells were pretreated with rCTGF and kahweol at one hour intervals and then stimulated with Ang II for 24 h. Protein levels of YAP and β-actin were determined by Western blotting with specific antibodies. Bar graphs present the densitometric quantification of the Western blot bands. (**C**) Cells were pretreated with verteporfin (100 nM) for one hour and then treated with Ang II (100 nM) for 24 h. Protein levels of phosphorylated FAK, YAP, CTGF and β-actin were determined by Western blotting with specific antibodies. Bar graphs present the densitometric quantification of the Western blot bands. Results are representative of three independent experiments. *, *p* < 0.05 vs. untreated control; #, *p* < 0.05 vs. Ang II group; §, *p* < 0.05 vs. kahweol + Ang II treated group.

Yes-associated protein (YAP) is known as a transcriptional regulator of the CTGF gene, and it has been reported that FAK controls durotaxis by hepatic stellate cells through YAP activation [30]. Thus, whether kahweol regulates Ang II-induced YAP expression was investigated. The result showed that Ang II increased YAP expression, and kahweol suppressed it, which was reversed by rCTGF administration (Figure 6A,B). Moreover, to demonstrate whether YAP contributes to CTGF and other protein expression in Ang II-stimulated VSMCs, YAP activity was inhibited by verteporfin, known as a YAP inhibitor. Ang II-induced FAK phosphorylation, YAP, and CTGF expression were significantly suppressed by verteporfin (Figure 6C). These data suggest that kahweol inhibits Ang II-induced YAP expression, and the YAP acts as a transcriptional regulator of Ang II-induced CTGF in VSMCs.

## 3. Discussion

Several studies have revealed that an increase in CTGF expression markedly enhances proliferation, migration, and ECM production in various cell types [19,20,22]. In previous studies, it was demonstrated that advanced glycation end product (AGE) and high glucose levels could induce the expression of CTGF via Erk and c-Jun *N*-terminal kinase (JNK) phosphorylation, respectively, resulting in the induction of proliferation, migration, and ECM production in VSMCs [26,31]. In the present study, Ang II increased CTGF expression in dose- and time-dependent manners (Figure 1A,B), while rCTGF increased ECM expression (Figure 4B) and migration (Figure 5C). Therefore, these results indicate that Ang II-induced CTGF expression promotes ECM production and migration in VSMCs.

Ang II induces synthetic VSMCs that have the capability to migrate from media to intima and to produce ECM proteins, contributing to artery wall thickness and stiffness in atherosclerosis [32,33]. It has been reported that CTGF, which is overexpressed in atherosclerotic plaques, induces blood mononuclear cell migration [27]. In addition, it has been demonstrated that CTGF is involved in oxidative stress-induced VSMC phenotype switching [29]. In the present study, it was demonstrated that Ang II-induced CTGF contributes only to synthetic marker expressions, such as those of fibronectin and collagen III, whereas SM22α and α-SMA, as contractile markers, are not affected. (Figure 2). To confirm whether regulation of synthetic phenotype by kahweol is CTGF dependent, exogenous rCTGF was used to induce CTGF protein expression [34]. rCTGF was administrated instead of Ang II-induced CTGF. The rCTGF treatment increased fibronectin expression, while the SM-22α and α-SMA contractile markers expressions were not affected (Figure 4B). These results suggest that synthetic phenotype marker switching is dependent on CTGF. According to previous studies, the expression interaction between synthetic and contractile markers tends to be inversely proportional [9,35]. However, it is unlikely that the expressions of both markers always have an inversely proportional relationship. In human aortic VSMCs, Branchetti et al. showed that CTGF significantly increased the expressions of synthetic markers collagen I, fibronectin, vimentin, and matrix metalloproteinase (MMP), while the expression of the contractile marker smoothelin B was not altered [29]. These observations support the suggestion that CTGF is involved in synthetic phenotype switching in VSMCs. Moreover, they imply that CTGF may be a molecular target for atherosclerosis in VSMCs.

Seo et al. reported that kahweol inhibits hepatic fibrosis by inhibiting the expression of CTGF via the TGF-β signaling pathway, including the involvement of STAT3, Smad3, Erk, and JNK [16]. Recently, it was demonstrated that kahweol acetate inhibits proliferation and migration of prostate cancer cells [36]. In the present study, Ang II-induced CTGF expression was significantly reduced by kahweol treatment (Figure 1C). In addition, inhibitory effects of kahweol on Ang II-induced CTGF-dependent synthetic marker expression (Figure 2) and migration (Figure 3C,D) were observed. These results suggest that kahweol inhibits CTGF-dependent synthetic phenotype switching and migration, thereby preventing the development of atherosclerosis in the intima. These findings imply that kahweol may be a useful agent in the prevention of atherosclerosis.

Chang et al. showed that Erk and Akt phosphorylation mediate serum-induced proliferation, and FAK and Erk phosphorylation mediate migration in PDGF-stimulated rat aortic VSMCs [9]. In the present study, it was observed that kahweol inhibits Ang II-induced FAK and Erk phosphorylation, and rCTGF reversed the kahweol effects (Figure 3A,B). However, only FAK phosphorylation was significantly affected by kahweol in Figure 5, whereas both FAK and Erk phosphorylation was reduced by kahweol in Figure 3. In our knowledge, unlike rCTGF stimulation, Erk inhibited by kahweol may be expressed through a pathway activated by Ang II stimulation. In Figure 6C, FAK phosphorylation was inhibited by verteporfin, as a YAP inhibitor. It could support that FAK phosphorylation is dependent on CTGF. Phosphorylation of FAK allows recruitment and activation of focal adhesion (FA) components including paxillin, vinculin, and Src in response to cell-ECM adhesion through integrins [37,38]. The data presented in Figure 3 suggest that kahweol inhibits VSMC migration by inhibiting CTGF through FAK and Erk phosphorylation. Wu et al. demonstrated that CTGF-activated fibroblasts promote tumor migration [39]. Liu et al. showed that FAK and Erk phosphorylation contribute to the migration of monocytes in human synovial fibroblasts [40]. These studies support the observation that FAK and Erk phosphorylation are involved in Ang II-induced VSMC migration. To clarify the underlying mechanism details of kahweol effect in Ang II-stimulated VSMCs, further studies on the detailed mechanism cascade are needed.

As shown in Figure 6, kahweol inhibited Ang II-induced YAP expression, and rCTGF reversed the kahweol effect. CTGF is known as a target gene of YAP [41,42], and Lin et al. showed that YAP has a pivotal role in Ang II-induced hypertensive vascular remodeling and phenotypic switching in VSMCs [43]. Lachowski et al. showed that FAK controls the mechanical activation of YAP, which is required for durotaxis in human hepatic stellate cells [30]. In addition, Nardone et al. demonstrated that YAP regulates cell mechanics by controlling focal adhesion assembly in adipose tissue derived mesenchymal stem cells and breast cancer cells [44], while Shen et al. showed that YAP promotes focal adhesion by activating FAK in breast cancer [45]. Moreover, Yu et al. reported that CTGF expression is induced through Erk-YAP signaling in taurocholate-stimulated hepatocytes [46]. These studies support the suggestion that YAP is involved as a transcriptional regulator for CTGF induction in Ang II-stimulated VSMCs, and kahweol inhibits CTGF expression through YAP regulation.

In conclusion, the current study describes the effects of kahweol on CTGF expression and synthetic phenotype switching. A key observation of this study is that kahweol inhibits CTGF-dependent synthetic phenotype marker expression and migration in Ang II-stimulated VSMCs. Additionally, kahweol regulates FAK, Erk, and YAP activation, which are increased in Ang II-stimulated VSMCs. Our study into the effects of kahweol on the induction of CTGF-dependent synthetic phenotype switching and migration in VSMCs may contribute to the development of treatments for atherosclerosis.

## 4. Materials and Methods

### 4.1. Reagents and Antibodies

The A7r5 cell line was bought from ATCC (Manassas, VA, USA). Recombinant human CTGF/CCN2 was purchased from R&D Systems (Minneapolis, MN, USA). Angiotensin II was purchased from Sigma-Aldrich (St. Louis, MO, USA). Kahweol was purchased from Santa Cruz Biotechnology (Dallas, TX, USA). Antibodies were purchased from the following vendors: Akt, Erk, FAK, phosphor-Akt, phosphor-Erk, and phosphor-FAK from Cell Signaling Technology (Danvers, MA, USA), CTGF, fibronectin, and YAP from Santa Cruz Biotechnology (Dallas, TX, USA), SM22α and α-SMA from Abcam (Cambridge, MA, USA), and β-actin from Sigma-Aldrich (St. Louis, MO, USA). Fetal bovine serum (FBS) was purchased from RMBIO (Missolula, MT, USA). Penicillin/streptomycin antibiotics and trypsin-EDTA solution were purchased from Hyclone (South Logan, UT, USA).

### 4.2. Primary Cell Culture and Drug Treatment

Sprague-Dawley rats at 6-week of age were sacrificed by dislocation of cervical spines. Primary rat vascular smooth muscle cells (VSMCs) were isolated from rat thoracic aorta. Thoracic aortas were isolated, placed in serum-free DMEM, and fatty tissues and adventitia removed. The vascular endothelium was mechanically removed by heavy pipetting with a 200 μL pipet tip and then cut into 3–5 mm long pieces. These pieces were explanted lumen side down on new 100 mm dishes containing DMEM supplemented with 50% FBS and incubated in a 5% CO_2_ air incubator for three days at 37 °C, after which, aorta pieces were removed and sprouted VSMCs were collected. These primary cells were maintained in DMEM with 10% FBS and 1% antibiotics (penicillin 10,000 U/mL, streptomycin 10,000 μg/mL). For experiments, we used VSMCs form passages 4–7 at 70–90% confluence. Cells in serum-free medium were treated with kahweol (10 µM) for one hour and then treated with Ang II (100 nM) for another 24 h.

### 4.3. Western Blot Analysis

After experimental treatments, cells were lysed with RIPA protein extract buffer supplemented with 10 μM protease inhibitor cocktail and 10 μM phosphatase inhibitor cocktail, and lysates were incubated on ice for 30 min and centrifuged at 15,000× *g* for 10 min at 4 °C Protein concentrations were determined using protein assay reagent from Bio-Rad (Hercules, CA, USA). Briefly, equal amounts of protein were mixed with Laemmli Sample Buffer (Bio-Rad) and heated for 5 min at 95 °C before loading. Protein samples were then separated by SDS-PAGE for 12 min at 60–90 V. Separated proteins were electrophoretically transferred to PVDF membranes (Bio Lab, Ubi, Singapore) for one hour at 100 V, and membraned were blocked with 5% non-fat milk in TBS containing 0.05% Tween 20 (TBS-T) for one hour at room temperature and then incubated overnight with primary antibodies (1:1000). Membranes were then washed in TBS-T and incubated for one hour at room temperature in 5% non-fat milk containing anti-rabbit or anti-mouse IgG. After washing with TBS-T, signals were visualized by using electrochemiluminescence (ECL) detection reagents (Millipore, CA, USA), according to the manufacturer’s instructions.

### 4.4. Quantitative Real-Time RT-PCR (qRT-PCR)

Total RNA was isolated by TRIzol (Thermo, MA, USA) and a reverse transcription (RT) reaction was conducted using the SuperScript^®^ III First-Strand Synthesis System for RT-PCR kit, following the manufacturer’s instructions. qRT-PCR was conducted with 1 μL of template cDNA and power SYBR Green using CFX connect real-time system (BIO RAD, CA, USA). Quantification was performed by the efficiency-corrected ΔΔCq method. The primers used to amplify the DNA sequences were as follows: CTGF (forward: 5′-GAGGAAAACATTAAGAAGGGCAAA-3′; reverse: 5′-CGGCACAGGTCTTGATGA-3′); Fibronectin (forward: 5′-GCTGCTGGGACTTCCACGT-3′; reverse: 5′-TCTGTTCCGGGAGGTGCA-3′); Collagen III (forward: 5′-GCGAAGGCAACAGTCGATTC-3′; reverse: 5′-CCCAAGTTCCGGTGTGACTC-3′); GAPDH (forward: 5′-GGAAAGCTGTGGCGTGAT-3′; reverse: 5′-AAGGTGGAAGAATGGGAGTT-3′). The PCR conditions were as follows: initial denaturation at 95 °C for 10 min, 95 °C for 10 s, 60 °C for 15 s, and 72 °C for 1 min.

### 4.5. Wound Scratch Assay and Transwell Chamber Assay

Cells were grown to 90% confluence in 6-well plates in DMEM supplemented with 10% FBS, and the medium was replaced with serum-free DMEM. Wounds were made with a sterile 200 μL pipet tip by drawing a line through the plated cells perpendicular to the line above. Cells were pretreated with kahweol (10 μM) in the presence or absence of recombinant CTGF (rCTGF) (50 ng/mL) for one hour and then exposed to Ang II (100 nM) for 48 h. Images were acquired by using a phase-contrast microscope (40×). To perform the transwell assay, cells were seeded in the inner chamber of the transwell plate and treated under the same conditions as described for the wound healing assay. At assay conclusion, cells were fixed by 4% paraformaldehyde and visualized by crystal violet staining. Non-migrated cells were scraped off with cotton swabs and then migrated cells were counted under a light microscope (OLYMPUS, Tokyo, Japan).

### 4.6. Statistical Analysis

Excel (Microsoft, Redmond, WA, USA) was used for data acquisition and analysis. Differences between data sets were assessed by one-way analysis of variance (ANOVA) or Bonferroni’s *t*-test. Results are presented as the means ± standard error of the mean (SEM) from at least three independent experiments, and *p* values of < 0.05 were considered significant.

## Sample Availability

Samples of the compounds are not available from the authors.
